# Titania Nanosheet Generates Peroxynitrite-Dependent *S-*Nitrosylation and Enhances p53 Function in Lung Cancer Cells

**DOI:** 10.3390/pharmaceutics13081233

**Published:** 2021-08-10

**Authors:** Rapeepun Soonnarong, Sucharat Tungsukruthai, Bodee Nutho, Thanyada Rungrotmongkol, Chanida Vinayanuwattikun, Tosapol Maluangnont, Pithi Chanvorachote

**Affiliations:** 1 Interdisciplinary Program of Pharmacology Graduate School, Chulalongkorn University, Bangkok 10330, Thailand; rapeepun.s@student.chula.ac.th; 2 Cell-Based Drug and Health Products Development Research Unit, Faculty of Pharmaceutical Sciences, Chulalongkorn University, Bangkok 10330, Thailand; 3 Department of Pharmacology, Faculty of Science, Prince of Songkla University, Hat Yai 90110, Songkhla, Thailand; sucharat.tungsukruthai@gmail.com; 4 Department of Pharmacology, Faculty of Science, Mahidol University, Bangkok 10400, Thailand; bodee.nut@mahidol.ac.th; 5 Biocatalyst and Environmental Biotechnology Reseach Unit, Department of Biochemistry, Faculty of Science, Chulalongkorn University, Bangkok 10330, Thailand; thanyada.r@chula.ac.th; 6 Program in Bioinformatics and Computational Biology, Graduate School, Chulalongkorn University, Bangkok 10330, Thailand; 7 Department of Medicine, Division of Medical Oncology, Chulalongkorn University, Pathumwan, Bangkok 10330, Thailand; chanida.Vi@chula.ac.th; 8 King Mongkut’s Institute of Technology Ladkrabang, College of Materials Innovation and Technology, Bangkok 10520, Thailand; tosapol.ma@kmitl.ac.th; 9 Department of Pharmacology and Physiology, Faculty of Pharmaceutical Sciences, Bangkok 10330, Thailand

**Keywords:** apoptosis, nanosheets, lung cancer, p53, *S*-nitrosylation, peroxynitrite, molecular dynamics

## Abstract

Metal nanomaterials can enhance the efficacy of current cancer therapies. Here, we show that Ti_0__.8_O_2_ nanosheets cause cytotoxicity in several lung cancer cells but not in normal cells. The nanosheet-treated cells showed certain apoptosis characteristics. Protein analysis further indicated the activation of the p53-dependent death mechanism. Transmission electron microscopy (TEM) and scanning electron microscopy (SEM) analyses revealed the cellular uptake of the nanosheets and the induction of cell morphological change. The nanosheets also exhibited a substantial apoptosis effect on drug-resistant metastatic primary lung cancer cells, and it was found that the potency of the nanosheets was dramatically higher than standard drugs. Ti_0__.8_O_2_ nanosheets induce apoptosis through a molecular mechanism involving peroxynitrite (ONOO^−^) generation. As peroxynitrite is known to be a potent inducer of *S*-nitrosylation, we further found that the nanosheets mediated the *S*-nitrosylation of p53 at C182, resulting in higher protein-protein complex stability, and this was likely to induce the surrounding residues, located in the interface region, to bind more strongly to each other. Molecular dynamics analysis revealed that *S*-nitrosylation stabilized the p53 dimer with a $\Delta G_{\mathrm{bind}}^{\mathrm{residue}}$ of <−1.5 kcal/mol. These results provide novel insight on the apoptosis induction effect of the nanosheets via a molecular mechanism involving *S*-nitrosylation of the p53 protein, emphasizing the mechanism of action of nanomaterials for cancer therapy.

## 1. Introduction

Lung cancer is one of the most common cancers worldwide, with the highest mortality rate. Although a number of crucial components in the fight against lung cancer have been elucidated, including small-molecule tyrosine kinase inhibitors and immunotherapy, which have led to unprecedented survival benefits in selected patients, the overall cure and survival rates for non-small cell lung cancer (NSCLC) remain low [1]. Therefore, new research discovering new drugs and therapies is essential to improve clinical outcomes. Tumor suppressor p53 is an essential regulatory molecule that is implicated in cell cycle arrest and plays a mediator role in apoptosis in response to stress [2]. A key attribute of the p53 response is p53 stabilization, which results in a rapid increase in p53 steady-state levels. Considerable evidence has indicated that p53 stabilization largely depends on post-translational events that disengage p53 from its proteasomal degradation [3]. In all cases, this includes a series of post-translational modifications, some of which are known to impact the interaction between p53 and the mouse double minute 2 (MDM2) protein, representing the major mechanism for controlling p53 stability [4]. The activation of p53 results in an increase in BH3-only proteins, promoting Bax/Bak oligomerization. The induction of pro-apoptotic signaling leads to the formation of mitochondrial pores, the release of cytochrome c into the cytosol, the activation of caspases, and finally cell apoptosis [5]. However, the upregulation of pro-survival proteins, including mammalian target of rapamycin (mTOR) and anti-apoptotic proteins of the Bcl-2 family, has been shown to play roles in apoptosis resistance [6].

p53, an important tumor suppressor protein, has been intensively investigated as its functions are critical for cancers. The functions of the p53 protein are tightly associated with its protein conformation. The active conformation of this protein is the tetrameric form via an interaction of the tetramerization domain (TD) on the p53 protein. Studies have pointed out that the tetramerization of p53 is critical for DNA binding and post-translational modification as well as for p53 stability [7]. Cysteine thiol groups on the p53 molecule have been highlighted as sensitive sites for protein modification, and it was shown that the DNA-binding affinity could be altered by thiol blocking agents [8]. Certain cysteine residue amino acid replacements inhibit the binding of p53 to DNA [9]. In addition, several lines of evidence have demonstrated that the oxidative modification of cysteine residues within p53 can also influence the protein’s activity and stability [10,11,12].

Nitric oxide (NO) is a key intercellular messenger synthesized from l-arginine in a reaction catalyzed by NO synthases (NOS). NO is recognized as an important signaling molecule for controlling practically all critical cellular functions, and it is also a strong mediator of cellular damage [13]. Interestingly, NO has been shown to regulate the expression of many genes, and this effect is exerted, in part, via S-nitrosylation [14]. S-nitrosylation of a certain side of transcription factors can modulate transcription factor activation, protein stability, and localization, as in the case of p53 and hypoxia-inducible factor-1α (HIF-1α) [15]. In addition, NO can react with superoxide (O_2_^−^) to form the much more powerful oxidant peroxynitrite (ONOO^−^), which is a key component that determines the contrasting roles of NO in physiology and pathology [16]. Many of the biological effects ascribed with NO are actually related to the intermediate peroxynitrite. Even though peroxynitrite is a powerful oxidant, it reacts at a moderately slow rate with most biological molecules and is able to reach cell membranes, in part, through anion channels [17]. This makes the biological and pathological insinuations of peroxynitrite much more interesting because it can have more delicate and specific actions on cells.

Nanotechnology is a research field that has wide implications in the fields of chemistry, engineering, biology, and medicine. Nanotechnology has several applications in cancer biology, especially in the development of novel treatments [18]. Nanosheets are a developing class of nanomaterial that are highly anisotropic and flexible [19]. Ti_0.8_O_2_ nanosheets are the two-dimensionality (2D) analog of TiO_2_ with potential anti-cancer stem cell activity [20]. However, to the best of our knowledge, the nanotoxicity and mechanism of Ti_0__.8_O_2_ nanosheets for specific site-targeting strategies in NSCLC have not yet been investigated. Consequently, this study aims to investigate the effects of Ti_0__.8_O_2_ nanosheets on the cytotoxicity of human non-small cell lung cancer (NSCLC) cells and to identify the molecular mechanisms behind the toxicity of these cells, which we reveal to be related to ROS generation-mediated apoptosis via the mitochondrial pathway. This study could be valuable in the development of nanomaterials for anti-cancer approaches. 

## 2. Materials and Methods

### 2.1. Ti_0__.8_O_2_ Nanosheet Synthesis and Characterization

The Ti_0__.8_O_2_ nanosheets were prepared as reported previously. Briefly, the potassium zinc titanate K_0__.8_Zn_0__.4_Ti_1__.6_O_4_ was first synthesized by heating the stoichiometric mixture of K_2_CO_3_, ZnO, and TiO_2_ at 900 °C for 20 h. Then, the solid was soaked in 1 M HCl overnight (solid-to-solution ratio of 1 g to 100 mL) for a total of 3 cycles, with fresh acid replaced in between. The product is H_1__.6_Ti_1__.6_O_4_·0.8H_2_O, where 0.8H^+^ is first exchanged for 0.8K^+^, another 0.8H^+^ for the leached [21] 0.4Zn^2^^+^, with water inclusion. Finally, 0.4 of the protonic form was mechanically shaken at 180 rpm for 14 days with diluted tetrabutylammonium hydroxide (TBAOH) solution (1 M, Sigma-Aldrich, St. Louis, MO, USA). The solid-to-solution ratio was fixed at 0.4 g-to-100 mL and the TBA^+^/H^+^ ratio at 1. The white colloid of Ti_0__.8_O_2_ nanosheets was then obtained.

The absorption characteristics of the nanosheets colloid were measured using a T90^+^ UV/VIS spectrometer (PG Instruments, Lutterworth, UK). The “size” of the nanosheets (i.e., the hydrodynamic radius, as determined by dynamic light scattering) and the zeta potential were measured using a Beckman Coulter Delsa Nano (Beckman Coulter Inc., Fullerton, CA, USA) instrument. The nanosheets were also imaged using a JEOL JEM 2010 (Beckman Coulter Inc., Fullerton, CA, USA) transmission electron microscope. Other results can be found in more detail elsewhere.

### 2.2. Cell Culture and Reagents

H23, H292, H460, and A549 lung cancer cells were obtained from the American Type Culture Collection (Manassas, VA, USA). A primary human dermal papilla cell line (primary DP1) was purchased from Celprogen (Benelux, The Netherlands). A human keratinocyte cell line (HaCaT) was purchased from Cell Lines Service (Heidelberg, Germany). Immortalized dermal papilla cells (DP) and human primary hair follicle dermal papilla cells (primary DP2) were purchased from Applied Biological Materials Inc (Richmond, BC, British Columbia). H460, H292, and H23 cells were cultured in RPMI 1640 medium (Gibco, Grand Island, NY, USA), whereas A549, HaCaT, DP, and primary DP1 and DP2 cells were cultivated in DMEM medium (Gibco, Grand Island, NY, USA). The medium was supplemented with 10% fetal bovine serum (FBS), 100 units/mL penicillin/streptomycin, and 2 mM L-glutamine (Gibco, Waltham, MD, USA). The cells were incubated in a 5% CO_2_ environment at 37 °C. Phosphate buffer saline (PBS) and trypsin-EDTA were purchased from GIBCO (Grand Island, NY, USA). Dimethyl sulfoxide, MTT, Hoechst 33342 staining, bovine serum albumin (BSA) dihydroethidium (DHE), and propidium iodide (PI) were purchased from Sigma Chemical, Inc. (St. Louis, MO, USA). An apoptosis kit (FITC) was purchased from ImmunoTools (Friesoythe, Germany). 2′,7′-Dichlorofluorescein (DCF) and 3′-p-(hydroxyphenyl) fluorescein (HPF) were purchased from Invitrogen (Waltham, MD, USA). Mn(III)tetrakis (4-benzoic acid) porphyrin (MnTBAP) was purchased from Merck (Calbiochem, La Jolla, CA, USA). Antibodies for p-p53 (Ser15), p53, Bcl-2, Mcl-1, Bax, caspase3, and β-actin, as well as peroxidase-conjugated secondary antibodies, were obtained from Cell Signaling Technology, Inc. (Danvers, MA, USA). Pierce *S*-nitrosylation Western blot kits were obtained from Thermo Fisher Scientific (Calbiochem, La Jolla, CA, USA).

### 2.3. Patient-Derived Primary Lung Cancer Cell Line Preparation from Malignant Pleural Effusion

The patient-derived lung cancer cells were prepared from pleural effusion of recurrent or advanced stage non-small cell lung cancer patients at the King Chulalongkorn Memorial Hospital. The Ethics Committee of the Faculty of Medicine, Chulalongkorn University, Bangkok, Thailand (IRB 581 365/62) approved the protocol. Informed consent from all contributors was obtained. Pleural effusion (approximately 1000 mL) was collected through thoracentesis. The samples were centrifuged at 300× *g* for 10 min. The cells were cultured in RPMI medium with 10% FBS, 2 mM L-glutamine, and 100 units/mL each of penicillin and streptomycin.

### 2.4. Cytotoxicity Assay

Cells were seeded onto 96-well plates at the density of 1 × 10^4^ cells/well and were allowed to incubate overnight. Then, cells were treated with various concentrations (0–100 μg/mL) of Ti_0__.8_O_2_ nanosheets for 24 h at 37 °C and analyzed for cell viability using a 3-(4,5-dimethylthiazol-2-yl)-2,5-diphenyltetrazolium bromide (MTT) assay. The MTT product was measured at 570 nm using a microplate reader. Cell viability was calculated by dividing the absorbance of the treated cells by that of the control cells and represented in percentage. All analyses were performed in 3 independent replicate cell cultures.

### 2.5. Nuclear Staining Assay

To determine apoptotic and necrotic cell death, H460 and A549 cells were seeded onto 96-well plates at the density of 1 × 10^4^ cells/well, incubated overnight, and then treated with Ti_0__.8_O_2_ nanosheets at various concentrations (0–10 μg/mL) for 24 h at 37 °C. After that, the cells were incubated with Hoechst 33342 (10 μg/mL) and propidium iodide (PI) (5 μg/mL) for 30 min at 37 °C in the dark. These cells were imaged under a fluorescence microscope (Nikon ECLIPSE Ts2, Tokyo, Japan).

### 2.6. Cell Apoptosis Analysis

The stages of apoptosis and necrosis cells were determined with annexin V-FITC apoptosis kits (ImmunoTools, Germany). H460 and A549 cells were seeded in 24-well plates at a density of 1 × 10^5^ cells/mL and treated with Ti_0__.8_O_2_ nanosheets at various concentrations (0–10 μg/mL) for 24 h. Then, the cells were detached and suspended in 100 μL of 1× binding buffer and incubated in 5 μL of annexin V-FITC and 1 μL of PI for 15 min at room temperature in the dark. Next, the cells were analyzed by guava easyCyte^TM^ flow cytometry systems.

### 2.7. Scanning Electron Microscopy (SEM) Morphological Analysis

Cells were fixed in 2.5% glutaraldehyde in 0.1 M phosphate buffer (pH 7.2) for 2 h. Next, the cells were dehydrated with a graded series of ethanol (30%, 50%, 70%, and 95% for 5 min/each and 100% 3 times, 5 min/time), dried, mounted, and coated with gold (sputter coater, Balzers model SCD 040, Wetzlar, Germany). Finally, the cells were observed under SEM (JEOL, model JSM6400, Tokyo, Japan).

### 2.8. Transmission Electron Microscopy (TEM) for Cellular Uptake Analysis

Cells were seeded at the density of 1 × 10^6^ cells/mL and treated with Ti_0.8_O_2_ nanosheets at 10 μg/mL for 24 h. The cells were collected, washed with PBS, and fixed in 2% glutaraldehyde, post-fixed in 1% osmium tetroxide, dehydrated in alcohol, and embedded. Thin sections of the resin-embedded cells were cut and subjected to a JEM-1400 (Jeol Ltd., Tokyo, Japan) transmission electron microscope (TEM).

### 2.9. Western Blot Analysis

After treatment, the cells were lysed, as previously described. Equal amounts of protein from each sample were subjected to SDS-PAGE for separation and transferred to nitrocellulose or PVDF membranes (Bio-Rad, Hercules, CA, USA). The blots were blocked for 1 h. with 5% non-fat dry milk [20] and, after that, incubated with specific primary antibodies against p-p53 (Ser15), p53, Bcl-2, Mcl-1, Bax, caspase 3, and β-actin at 4 °C overnight. Then, the blots were washed in TBST and incubated with horseradish peroxidase (HRP)-conjugated secondary antibodies for 2 h at room temperature. Finally, protein bands were detected using an enhanced chemiluminescence substrate (Supersignal West Pico; Pierce, Rockford, IL, USA) and exposed to film.

### 2.10. ROS, Superoxide Anion, and Hydroxyl Radical Detection by Flow Cytometry

A549 and H460 were seeded in 24-well plates and cultured for 12 h. DCFHDA, DHE, and HPF were added, and the cells were incubated for 30 min in the dark. Cells were treated with Ti_0.8_O_2_ nanosheets for 3 h, washed, and resuspended in PBS. DCF fluorescence was quantified using guava easyCyte^TM^ flow cytometry systems.

### 2.11. NO Detection by DAF-FM DA Assay

After detachment, the cells were collected and incubated with 10 µM DAF-FM DA for 30 min at 37 °C. The cells were then washed, resuspended in phosphate-buffered saline, and analyzed for fluorescence intensity using guava easyCyte^TM^ flow cytometry systems. These cells were imaged under a fluorescence microscope (Nikon ECLIPSE Ts2).

### 2.12. Immunofluorescence

Cells were seeded onto 96-well plates at the density of 1 × 10^5^ cells/well. The cells were treated with a nanosheet and fixed with 4% (*w*/*v*) paraformaldehyde for 30 min. The cells were permeabilized with 0.1% (*v*/*v*) Triton-X for 20 min, incubated with 3% (*w*/*v*) BSA for 30 min, and washed and incubated with a p53 or p-p53 antibody overnight at 4 °C. The cells were washed and incubated with Alexa Flour 488 conjugated secondary antibodies for 1 h at room temperature. The cells were washed with PBS, co-stained with 10 μg/mL Hoechst 33342, and visualized under a fluorescence microscope (Nikon ECLIPSE Ts2).

### 2.13. Cycloheximide (CHX) Chasing Assay

Cells were seeded and treated with 10 μg/mL of Ti_0__.8_O_2_ nanosheets with or without 50 µg/mL CHX for 0, 15, 30, 45, 60, and 90 min. The cells were lysed with RIPA lysis buffer containing the protease inhibitor cocktail (Roche Diagnostics, Indianapolis, IN, USA). Western blot analysis was performed for detecting p53 protein levels. Protein bands were analyzed using ImageJ software (version 1.52, National Institutes of Health, Bethesda, MD, USA), and the Mcl-1 protein half-life was calculated.

### 2.14. Immunoprecipitation Assay

Cells were treated with Ti_0.8_O_2_ nanosheets for 1 h. Immunoprecipitation was performed using Dynabeads™ Protein G immunoprecipitation kits from Thermo Fisher Scientific Inc (Waltham, MA, USA). Magnetic beads were prepared and incubated with the p53 antibody for 20 min. A bead-Ab complex was mixed with cell lysate and incubated at 4 °C overnight. The bead-Ab-Ag complex was washed three times, separated on a magnet between each wash, and the supernatant was removed. Elution buffer was added. The supernatant was then subjected to Western blot analysis.

### 2.15. S-Nitrosylated Protein Detection

Cells were lysed and centrifuged at 10,000× *g* for 10 min. To each sample, 2 μL of 1 M MMTS was added, and the samples were incubated for 30 min at room temperature to block free cysteine thiols. Then, the protein was precipitated by adding pre-chilled (−20 °C) acetone and freezing at −20 °C to remove MMTS for at least 1 h. Samples were centrifuged at 10,000× *g* for 10 min at 4 °C. Pellets were resuspended in 100 μL of HENS buffer, 1 μL of the labeling reagent, and 2 μL of 1M sodium ascorbate for 1–2 h at room temperature. Finally, labeled-protein SDS-PAGE and Western blotting analysis were used to detect *S*-nitrosylation p53 proteins.

### 2.16. Computational Method

The X-ray structure of the tetrameric p53 core domain was taken from the Protein Data Bank (PDB ID: 3KMD) [22]. The H++ web server [23] was used to assign the protonation state of all ionizable groups of amino acids at pH 7.4. The modeled protein was then submitted to all-atom molecular dynamics (MD) simulations using the AMBER16 software package according to standard procedures [24,25,26], as summarized below. In brief, the starting structure of the p53 protein was firstly energy-minimized using the steepest descent (500 steps) and a conjugated gradient (1500 steps) based on the ff14SB AMBER force field [27] to reduce unfavorable contacts. After that, a 100-ns MD simulation with the NPT ensemble at 310 K and 1 atm was carried out by the PMEMD module of AMBER16. The SHAKE algorithm [28] was applied to restrain the covalent bond involved in hydrogen atoms, allowing a simulation time step of 2 fs. The particle mesh Ewald [29] summation method was used to treat the long-range electrostatic interactions, whereas a nonbonded cutoff distance was set to 10 Å. The MD trajectories in the production phase were collected every 10 ps and analyzed in terms of intermolecular hydrogen bonding interaction using the CPPTRAJ module [30] of AMBER 16. To determine the essential residues associated with protein–protein binding at the interface of the four monomers and the effect of *S*-nitrosylation on the cysteine (Cys) residue towards protein stability, the per-residue decomposition free energy ($\Delta G_{\mathrm{bind}}^{\mathrm{residue}}$) method, based on the molecular mechanics/Poisson-Boltzmann surface area (MM/PBSA), was performed using the MMPBSA.py module [31] implemented in AMBER16.

### 2.17. Statistical Analysis

Data from three independent experiments are presented as mean ± standard error of mean (SEM). Multiple comparisons for statistically significant differences between multiple groups were performed using analysis of variance (ANOVA), followed by Turkey’s post hoc test. *p*-values < 0.05 were considered statistically significant.

## 3. Results

### 3.1. Synthesis and Characterization of the Ti_0__.8_O_2_ Nanosheets

**Figure 1:**
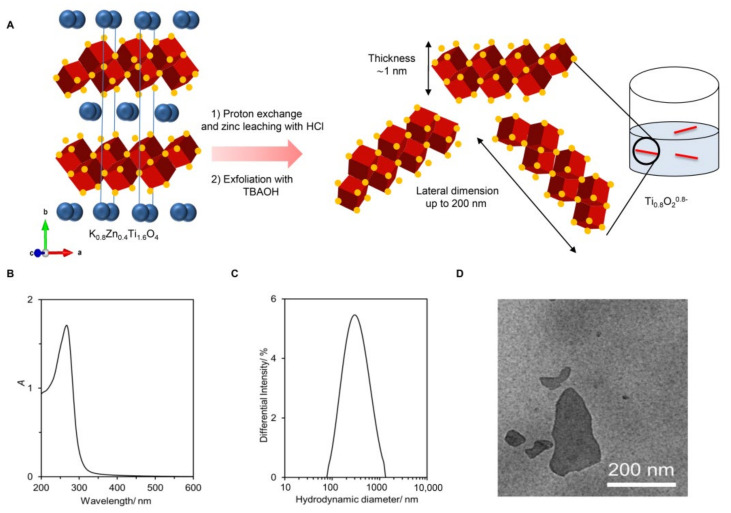
Synthesis and characterization of the Ti_0__.8_O_2_ nanosheets. (**A**) The schematic diagram of Ti_0__.8_O_2_ nanosheet preparation. The crystal structure of K_0__.8_Zn_0__.4_Ti_1__.6_O_4_ shows K^+^ ions (green) sandwiched between the double edge-shared O atoms in red and Ti atoms at the center in blue; (**B**) UV-vis absorption spectrum and photograph of the nanosheets without and with the laser light shining through; (**C**) size of the nanosheets; (**D**) representative TEM image of the deposited nanosheets.

The preparation of the Ti_0__.8_O_2_ nanosheets from lepidocrocite-type K_0__.8_Zn_0__.4_Ti_1__.6_O_4_ is shown schematically in Figure 1A. Here, the K^+^ ion (0.8 per the formula unit) alternately stacks with the negatively-charged sheets of edge-shared (Ti, Zn)O_6_ octahedra (i.e., the Zn_0__.4_Ti_1__.6_O_4_^0^^.8^^−^ sheet with ~1 nm thickness). Reacting the solid with 1 M HCl led to a quantitative replacement of K^+^ with H_3_O^+^ and almost complete Zn leaching. The subsequent reaction of the solid with the bulky TBA^+^ ions resulted in the infinite separation of stacks of sheets into individual nanosheets.

As shown in Figure 1B, the obtained colloidal suspension was white with a blue tint. It absorbed light at λmax = 261 nm, which is consistent with previous reports. A clear Tyndall effect could be observed, where laser illumination was scattered throughout, suggesting the presence of nanosheets. Dynamic light scattering provided the size estimation of the Ti_0__.8_O_2_ nanosheets as ~268 nm (see Figure 1C). The zeta potential of −30 mV agreed well with their negatively charged nature. Figure 1D is a representative TEM image, showing several uniform-contrast, flat objects (i.e., nanosheets) with lateral dimensions of up to ~200 nm. Altogether, the different characterization techniques confirmed the successful preparation of negatively charged Ti_0__.8_O_2_^0^^.8^^−^ nanosheets (or simply Ti_0__.8_O_2_ nanosheets) in contrast to typical TiO_2_, which is charge-neutral. Detail characterizations can be found elsewhere [20].

### 3.2. Cytotoxicity of the Ti_0__.8_O_2_ Nanosheets on Human Lung Cancer Cells and Normal Cells

**Figure 2:**
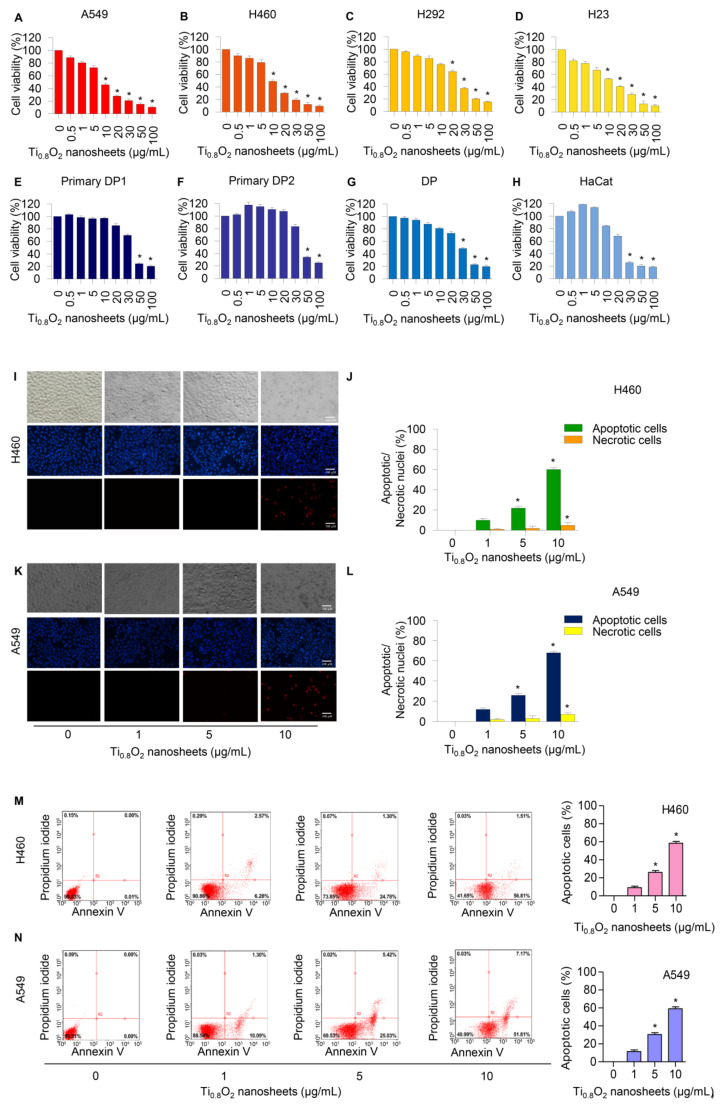
Cytotoxicity of Ti_0__.8_O_2_ nanosheets on human lung cancer cells and normal cells. Ti_0__.8_O_2_ nanosheets reduced cell viability and induced apoptosis in lung cancer cells. (**A**–**H**) Effect of Ti_0__.8_O_2_ nanosheets on cell viability of lung cancer cells (A549, H460, H292, and H23 cells) and normal cells (dermal papilla (DP), primary DP1, primary DP2, and HaCaT keratinocyte cells) for 24 h using MTT assays; (**I**–**L**) morphology of apoptotic nuclei stained with Hoechst 33342 dye and propidium iodide in cells treated with Ti_0__.8_O_2_ nanosheets, determined by visualization using fluorescence microscopy and calculated percentages of nuclear fragmented and PI-positive cells; (**M**,**N**) apoptotic and necrotic cells were determined using annexin V-FITC/PI staining with flow cytometry; data are shown as the mean ± SEM (*n* = 3). * *p* < 0.05 versus non-treated control.

Cells were treated with various concentrations of Ti_0__.8_O_2_ nanosheets (0–100 µg/mL) and analyzed by MTT assay. The results revealed the statistically significant cytotoxic effects of the Ti_0__.8_O_2_ nanosheets occurred at concentrations of 10–100 μg/mL in A549, H460, and H23 cells and at 20–100 μg/mL in H292 cells. In the primary dermal papilla cells from different sources (DP1 and DP2), cytotoxic effects of the Ti_0__.8_O_2_ nanosheets were found at 50 μg/mL. Moreover, at the concentration of 30 μg/mL, the Ti_0__.8_O_2_ nanosheets showed statistically significant cytotoxic effects on DP and HaCat cells (Figure 2A–H). Characteristic apoptosis cells were identified using a nuclear staining assay. The results showed that the Ti_0.8_O_2_ nanosheets mediated apoptosis in lung cancer cells at concentrations of 1–10 μg/mL, with a small percentage of necrotic cells (Figure 2I–L). Flow cytometry analysis based on annexin V/PI detection also confirmed that 10 μg/mL of Ti_0__.8_O_2_ nanosheets induced dramatic apoptosis in A549 and H460 cells compared with untreated cells (Figure 2M,N).

### 3.3. Uptake of the Ti_0__.8_O_2_ Nanosheets by Cancer Cells

**Figure 3:**
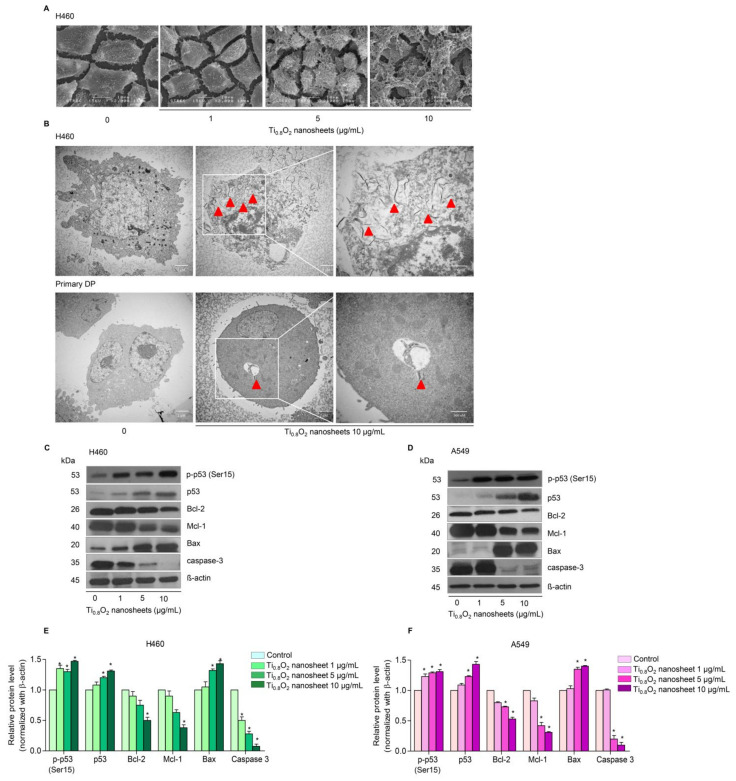
Morphology and characterization of cells when treated with Ti_0.8_O_2_ nanosheets. (**A**) Morphology of H460 cells determined by scanning electron microscopy (SEM); (**B**) cellular uptake of Ti_0.8_O_2_ nanosheets in H460 and primary DP cells at 24 h, determined by transmission electron microscopy (TEM); (**C**,**D**) effect of Ti_0.8_O_2_ nanosheets on apoptosis-related proteins measured by Western blot analysis. Blots were reprobed with β-actin to confirm the equal loading of samples; (**E**,**F**) relative protein levels, calculated by densitometry. Data are shown as the mean ± SEM (*n* = 3). * *p* < 0.05 versus non-treated control.

Under SEM morphological analysis, it was seen that for H460 cells, the morphology of the cancer cells gradually changed when treated with Ti_0__.8_O_2_ nanosheets at concentrations of 1–10 μg/mL (Figure 3A). Moreover, TEM analysis showed that Ti_0__.8_O_2_ nanosheets at 10 μg/mL could appropriately pass into the H460 cells (Figure 3B).

### 3.4. Ti_0__.8_O_2_ Nanosheets Modulate Apoptosis-Related Proteins in H460 and A549 Cells

In order to investigate the mechanism of Ti_0__.8_O_2_ nanosheet-induced apoptosis, the apoptotic-related proteins were determined by Western blot analysis. A549 and H460 cells were treated with 0–10 μg/mL Ti_0__.8_O_2_ nanosheets, and then the pro-and anti-apoptotic proteins related to mitochondria-mediated apoptosis were evaluated. The results showed that the Ti_0__.8_O_2_ nanosheets increased the pro-apoptotic protein Bax, whereas the anti-apoptotic proteins Mcl-1 and Bcl-2 were downregulated in the cells treated with the Ti_0__.8_O_2_ nanosheets. In addition, pro-caspase3 decreased in a concentration-dependent manner. Moreover, Ti_0__.8_O_2_ nanosheets caused a significant increase in both p-p53 (Ser15) and total p53 protein levels in a dose-dependent manner (Figure 3C–F). These findings suggest that p53 may play a role in Ti_0__.8_O_2_ nanosheet-induced apoptosis of NSCLC cells. Taken together, it can be concluded that the Ti_0__.8_O_2_ nanosheets mediated the apoptosis of lung cancer cells by increasing pro-apoptotic proteins, which led to cell death by the mitochondria-dependent pathway.

### 3.5. Cytotoxicity and Apoptotic Effects of Ti_0__.8_O_2_ Nanosheets on Advanced Lung Cancer Cells from Patients

**Figure 4:**
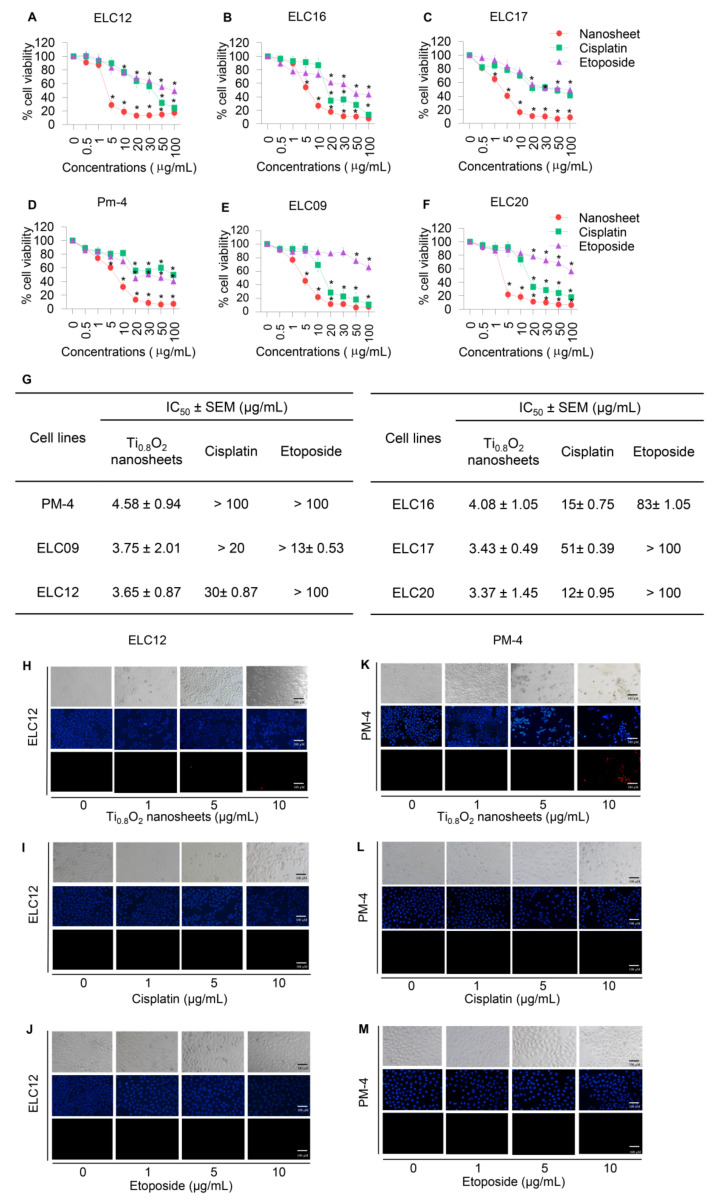
Cytotoxicity of the Ti_0__.8_O_2_ nanosheets on malignant pleural effusion from advanced lung cancer patients. (**A**–**F**) Effect of the Ti_0__.8_O_2_ nanosheets on the cell viability of malignant pleural effusion for 24 h using an MTT assay to determine the IC_50_ values; (**G**) percentages of cell viability were determined using an MTT assay; (**H**–**M**) morphology of apoptotic nuclei stained with Hoechst 33342 dye and propidium iodide in cells treated with Ti_0__.8_O_2_ nanosheets; cisplatin and etoposide were determined by visualization under a fluorescence microscope; the percentages of nuclear fragments and PI-positive cells were calculated. Data are shown as the mean ± SEM (*n* = 3). * p < 0.05 versus non-treated control.

To assess the potential pharmacological activities of synthetic Ti_0__.8_O_2_ nanosheet compounds in advanced lung cancer cells, treatment with current standard therapeutic agents was also performed for comparison. Two groups of cell lines were used for the investigations: Panel A, an advanced non-small cell lung cancer cell line from patients with malignant pleural effusion who had never been treated by chemotherapy, targeted therapy, or immunotherapy, and Panel B, an advanced non-small cell lung cancer cell line from patients with malignant pleural effusion who had been treated with standard platinum-doublet chemoRx with or without targeted therapy or a checkpoint inhibitor and second-line chemoRx. In total, six primary lung cancer cells were treated with the same concentrations of Ti_0__.8_O_2_ nanosheets, cisplatin, and etoposide (0–100 μg/mL) for 24 h and subjected to cell viability analysis by MTT assay. The Ti_0__.8_O_2_ nanosheets could be considered nontoxic at doses lower than 1 μg/mL, while a concentration of more than 10 μg/mL caused a significant decrease in the cell viability of the cells (Figure 4A–F). In contrast, the standard drugs showed slightly decreased cell viability from 0.5 to 100 μg/mL, while doses of more than 20 μg/mL cisplatin and etoposide were considered toxic. Data analysis showed that the IC_50_ of the Ti_0__.8_O_2_ nanosheets was lower than 10 μg/mL at 24 h, which was significantly lower than for cisplatin and etoposide (Figure 4G). The results showed that the Ti_0__.8_O_2_ nanosheets reduced cell viability in a concentration-dependent manner compared with the untreated controls (Figure 4A–F). To confirm the effect of the Ti_0__.8_O_2_ nanosheets on advanced lung cancer cells from patients, a nuclear staining assay using Hoechst 33342 and propidium iodide was performed and the results analyzed. After treatment with the compounds at 10 μg/mL of Ti_0__.8_O_2_ nanosheets, apoptotic cells were observed by the presence of nuclear condensation morphology in the representative cell line (Figure 4H–M). It was found that the potency of the nanosheets was dramatically higher than that of cisplatin and etoposide.

### 3.6. Effect of Ti_0__.8_O_2_ Nanosheets on Intracellular ROS Induction in A549 and H460 Cells

**Figure 5:**
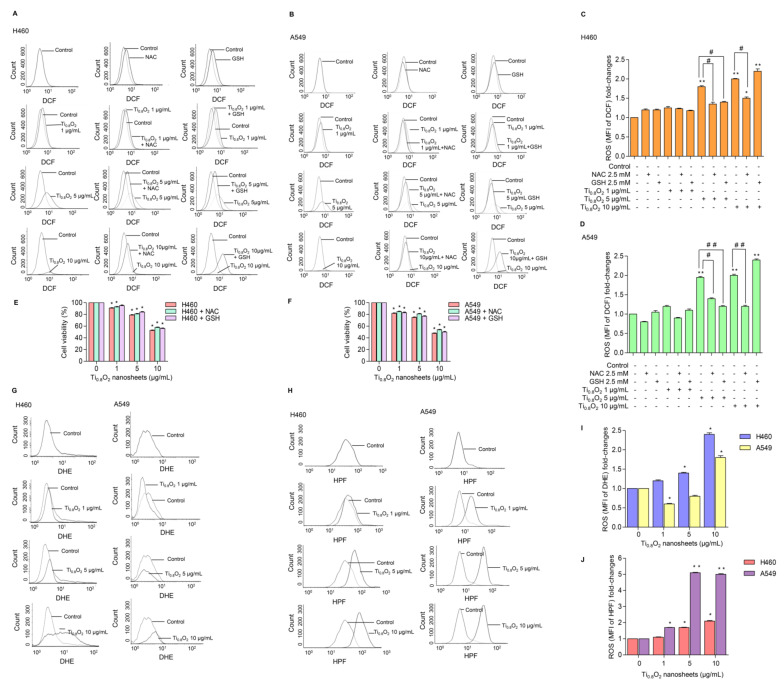
Ti_0__.8_O_2_ nanosheets induced intracellular ROS in H460 and A549 cells. (**A**–**D**) The effect of Ti_0__.8_O_2_ nanosheets (0–10 g/mL) on intracellular ROS induction at 24 h in H460 and A549 cells was determined by flow cytometry with the fluorescent probe DCF (10 µM). Cells were treated with Ti_0__.8_O_2_ nanosheets (0–10 µg/mL) alone for 24 h or with the pretreatment of 2.5 mM NAC and 2.5 mM GSH. ROS levels were calculated and presented as the mean fluorescence intensity (MFI); (**E**,**F**) effect of Ti_0__.8_O_2_ nanosheets on cell viability in H460 and A549 cells at 24 h with the pretreatment of 2.5 mM NAC or 2.5 mM GSH was determined by an MTT assay. Data are shown as the mean ± SEM (*n* = 3); (**G**,**I**) the effect of Ti_0__.8_O_2_ nanosheets (0–10 µg/mL) on superoxide anion induction at 24 h in H460 and A549 cells was determined by flow cytometry with the fluorescent probe DHE (10 µM). Superoxide anion levels were presented as mean fluorescence intensity (MFI); (**H**,**J**) the effect of Ti_0__.8_O_2_ nanosheets (0–10 µg/mL) on hydroxyl radical induction at 24 h in H460 and A549 cells was determined by flow cytometry with the fluorescent probe HPF (10 µM). Hydroxyl radical levels were calculated and indicated as the mean fluorescence intensity (MFI). All data are shown as the mean ± SEM (*n* = 3). * *p* < 0.05, ** *p* < 0.01 compared with the untreated control, and ^#^ *p* < 0.05, ^##^ *p* < 0.01 compared with the treated cells at the same concentration of Ti_0.8_O_2_ nanosheets.

ROS has been considered an important mediator of the initiation and execution of apoptosis and is related to the anti-cancer effect of several drugs [32]. We next investigated whether intracellular ROS generation was implicated in the anti-cancer effects of the Ti_0__.8_O_2_ nanosheets. The intracellular ROS level was evaluated using the fluorescent probe DCFH-DA. The results showed that treatment with Ti_0__.8_O_2_ nanosheets increased intracellular ROS generation (Figure 5A–D). In order to investigate the protective effect of N-acetylcysteine (NAC) or glutathione (GSH) as a potent antioxidant on Ti_0__.8_O_2_ nanosheet-induced cytotoxicity mediated through ROS generation, the H460 and A549 cell lines were pretreated with NAC or GSH for 1 h previous to treatment with the Ti_0__.8_O_2_ nanosheets. We detected a decrease in the ROS level in all the cell lines treated with NAC and GSH (Figure 5A–D), but the cell viability of the cancer cells could not be reversed by the pretreatment with NAC or GSH (Figure 5E,F). These results suggest that the Ti_0__.8_O_2_ nanosheets induce cytotoxicity in cancer cell lines but do not do this via the generation of ROS. Next, we investigated the specific ROS products using a DHE (dihydroethidium) fluorescent probe for the detection of ROS generation and, specifically, the detection of superoxide anions. The results showed that the Ti_0__.8_O_2_ nanosheets had a significant effect on the superoxide anions in H460 cells when they were treated with Ti_0__.8_O_2_ nanosheets in a concentration-dependent manner (Figure 5G,I), while the Ti_0__.8_O_2_ nanosheets had only a slight effect on superoxide anion generation in A549 cells (Figure 5E). In addition, we also investigated the generation of hydroxyl radicals using the HPF (hydroxyphenyl fluorescein) fluorescent probe in both cell lines. The results showed that the Ti_0__.8_O_2_ nanosheets significantly generated hydroxyl radicals in both cell lines compared with the non-treated cells (Figure 5H,J). According to our obtained data, the pretreatment of cancer cell lines with a potent antioxidant for 1 h could not inhibit H_2_O_2_ damage, while the Ti_0__.8_O_2_ nanosheets generated superoxide anion hydroxyl radicals in both cell lines.

### 3.7. Ti_0__.8_O_2_ Nanosheet-Mediated Peroxynitrite Induces Apoptosis in A549 and H460 Cells

**Figure 6:**
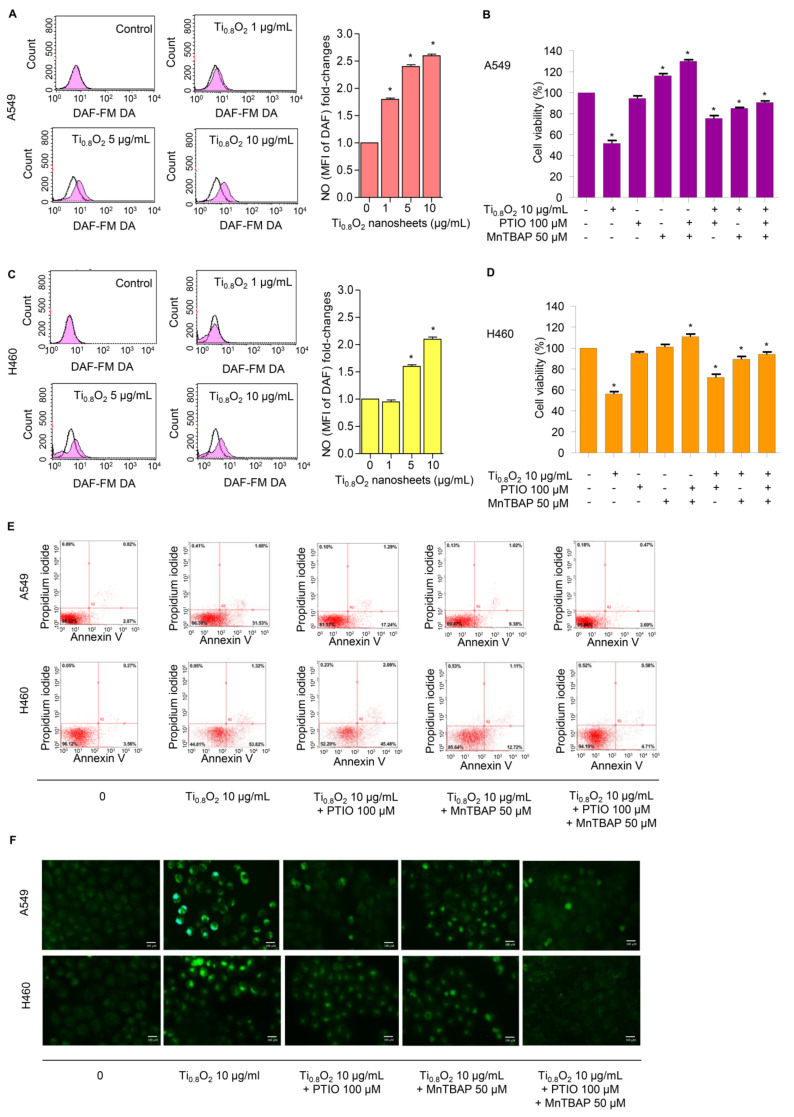
Ti_0.8_O_2_ nanosheets induced NO in A549 and H460 cells and mediated peroxynitrite generation in these cells. (**A**,**C**) Effect of Ti_0.8_O_2_ nanosheets on NO levels in A549 and H460 cells. Cells were treated with DAF-FM DA and various concentrations of Ti_0.8_O_2_ nanosheets (0–10 µg/mL) for 30 min, and the peroxynitrite levels were determined at 3 h by flow cytometry. NO levels are presented as mean fluorescence intensity (MFI); (**B**,**D**) the cytotoxic effect of Ti_0.8_O_2_ nanosheets and the NO scavenger (PTIO) on A549 and H460 cells. Effect of Ti_0.8_O_2_ nanosheets and the NO scavenger (PTIO) on cell viability. Cells were treated with Ti_0.8_O_2_ nanosheets (10 µg/mL) in the presence or absence of pretreatment with a NO scavenger (PTIO) (100 µM) or pretreatment with a superoxide inhibitor (MnTBAP) (50 µM) for 24 h by MTT assay; (**E**) apoptotic and necrotic cells were determined using annexin V-FITC/PI staining with flow cytometry; (**F**) cellular NO level stained with DAF-FM DA in cells treated with Ti_0.8_O_2_ nanosheets (10 µg/mL) or pretreated with PTIO (100 µM) or MnTBAP (50 µM) were determined by visualization under a fluorescence microscope. All data are shown as the mean ± SEM (*n* = 3). * *p* < 0.05 compared with the untreated control.

NO plays a role in apoptosis regulation through its ability to modulate ROS. The cytotoxic action of NO was demonstrated in several systems using diverse cell targets. Interestingly, the cytotoxicity of NO was shown to mediate via the interaction of NO with superoxide to form a highly reactive peroxynitrite (ONOO^−^) [33]. Consequently, we analyzed the cellular NO levels in response to the Ti_0__.8_O_2_ nanosheets using DAF-FM DA as a fluorescent probe. The NO levels were found to be increased in a concentration-dependent manner (Figure 6A,C). Additionally, co-treatment with PTIO (a NO scavenger) and/or MnTBAP (a superoxide anion inhibitor) inhibited Ti_0__.8_O_2_ nanosheet-induced cell death by increasing cell viability (Figure 6B,D). The results suggest that Ti_0__.8_O_2_ nanosheets induce cytotoxicity in cancer cell lines via the produced peroxynitrite. To confirm the previous results, we treated cells with Ti_0__.8_O_2_ nanosheets and/or pretreated them with PTIO and/or MnTBAP and then determined the cellular NO level by staining with DAF-FM DA and then visualization under a fluorescence microscope. The results showed that the co-treatment with these inhibitors decreased peroxynitrite levels in all the cancer cells (Figure 6F). Then, we observed whether increased peroxynitrite was required for cell apoptosis induced by Ti_0__.8_O_2_ nanosheets. The results showed that the co-treatment with these inhibitors was able to inhibit apoptosis cell death, as shown in Figure 6E. Collectively, these results indicated that peroxynitrite generation could play a role in mediating Ti_0__.8_O_2_ nanosheet-induced cell apoptosis.

### 3.8. Ti_0__.8_O_2_ Nanosheet-Mediated Peroxynitrite Induces Apoptosis in A549 and H460 Cells via p53 Upregulation

**Figure 7:**
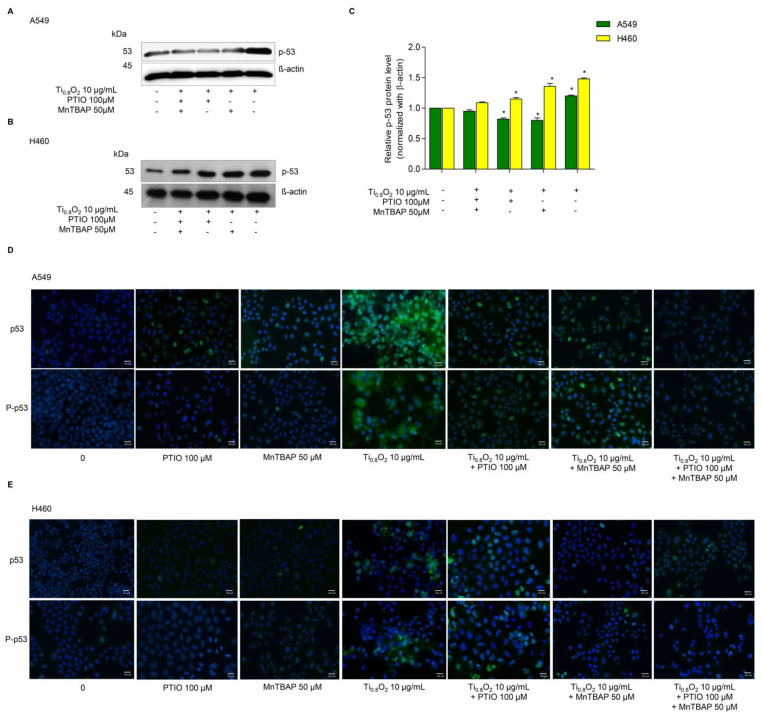
Ti_0__.8_O_2_ nanosheets associated with apoptosis in A549 and H460 cells via p53 upregulation. (**A**,**B**) Peroxynitrite-potentiated cell apoptosis through the p53 protein was measured by Western blot analysis; (**C**) blots were reprobed with β-actin to confirm the equal loading of samples. The relative protein levels were calculated by densitometry. Data are shown as the mean ± SEM (*n* = 3). * *p* < 0.05 versus non-treated control. (**D**,**E**) the expressions of p53 and P-p53 were analyzed by immunofluorescence staining in A549 and H460 cells.

Since then, several cell types have been shown to undergo apoptosis in response to NO or peroxynitrite. A previous study reported that peroxynitrite was associated with p53 regulation to induce cancer cell death [33]. Therefore, we examined the effect of Ti_0__.8_O_2_ nanosheets when combined with PTIO and/or MnTBAP. Western blot analysis was performed to evaluate the p53 protein levels after 10 µg/mL Ti_0__.8_O_2_ nanosheet treatment in all the cell lines. The results showed that the p53 protein levels in all the cell lines were significantly increased with the Ti_0__.8_O_2_-nanosheets-alone treatment compared with those of the non-treatment control and another condition treatment (Figure 7A–C). Taken together, it could be concluded that the Ti_0__.8_O_2_ nanosheets induced cancer cell death by the induction of peroxynitrite generation, which activated p53, leading to cancer cell apoptosis. The immunofluorescence staining results supported our finding that Ti_0__.8_O_2_ nanosheets, in combination with PTIO and MnTBAP, caused a dramatic decrease in the levels of p53 and P-p53 in both cell lines (Figure 7D,E). These results indicate that the pro-apoptotic effect of Ti_0__.8_O_2_ nanosheets for inducing NO is a result of the formation of peroxynitrite, which then induces p53-dependent apoptosis in all the cell lines.

### 3.9. Ti_0.8_O_2_ Nanosheets Increase p53 Function but Not through p53 Proteasomal Degradation

**Figure 8:**
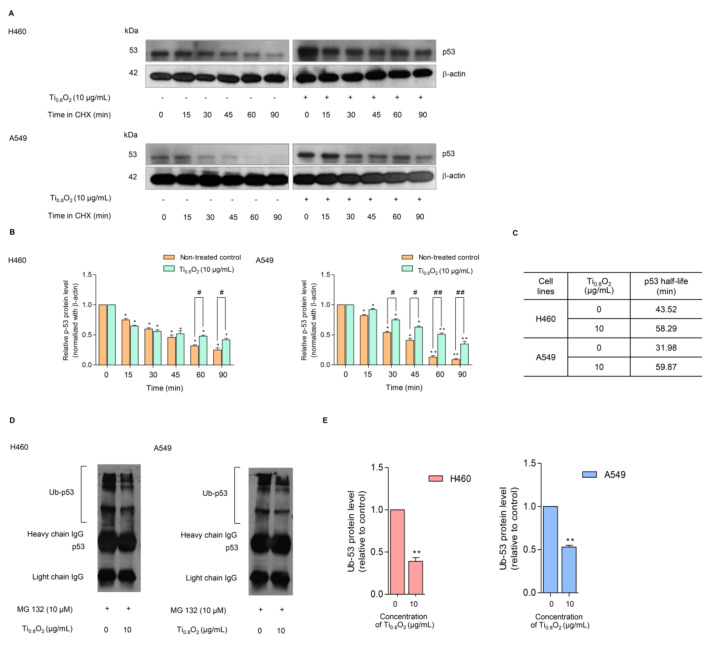
Effect of Ti_0__.__8_O_2_ nanosheets on the p53 level and proteasomal degradation. (**A**) The cycloheximine (CHX) chasing assay was performed to determine the half-life of p53. H460 and A549 cell lines were treated with 50 µg/mL of CHX with or without 10 µg/mL Ti_0__.__8_O_2_ nanosheets. Western blot analysis was used to determine the p53 protein level; (**B**) the relative p53 protein levels were calculated and compared with the control group at 0 min; (**C**) the half-lives of the p53 protein of H460 and A549 cells were calculated; (**D**) H460 and A549 cells were pretreated with 10 µM MG132 for 30 min, followed by treatment with 10 µg/mL Ti_0__.__8_O_2_ nanosheets for 60 min. The protein lysates were collected, and the p53 protein was pulled down using p53 antibodies. The levels of the ubiquitin-p53 protein complex were determined by Western blot analysis.; (**E**) p53-ubiquitin complexes were quantified by densitometry. The relative protein levels are reported (*n* = 3). * *p* < 0.05, ** *p* < 0.01 compared with the untreated control at 0 min, and ^#^ < 0.05, ^##^ *p* < 0.01 compared with the untreated control at the same time.

In general, p53 functions as a tumor suppressor protein in response to oncogenic stress signals [34]. The essential role of p53 is emphasized by the fact that this protein is commonly deregulated or altered in human malignancies [35,36]. The activation of p53 initiates a variety of cellular responses that stop the cell cycle, induce senescence, and activate apoptosis. The key to the regulation of p53 is the control of its stability, which is mainly arranged through a network of ubiquitination reactions. We further evaluated the effect of the Ti_0__.8_O_2_ nanosheets on p53 protein stability using the cycloheximide (CHX) chasing assay. CHX can inhibit protein biosynthesis and is widely used to investigate the half-life of proteins [37]. Therefore, H460 and A549 cells were treated with Ti_0.8_O_2_ nanosheets in the presence or absence of CHX, and the level of p53 over time was determined. Figure 8A,B shows that in the condition where protein production was blocked, the Ti_0__.8_O_2_ nanosheets increased the stability of the p53 protein. A difference was first detected at 90 min after Ti_0__.8_O_2_ nanosheet treatment (Figure 8A,B). We also determined the p53 protein’s half-life and found that the half-life of the p53 protein was approximately 60 min in the Ti_0__.8_O_2_ nanosheet-treated cells; In contrast, in the non-treated control cells, the half-life was about 30–40 min (Figure 8C).

Moreover, the turnover of the p53 protein is tightly regulated by ubiquitin–proteasome degradation. Thus, we used the specific proteasome inhibitor (MG132) to investigate whether the Ti_0.8_O_2_ nanosheets increased p53 stability via the inhibition of proteasomal degradation. Co-immunoprecipitation was also used to test the premise of ubiquitin-mediated p53 degradation in H460 and A549 cells after treatment with 10 µg/mL of Ti_0__.8_O_2_ nanosheets and in non-treated control cells for 1 h. Figure 8D,E shows that the polyubiquitination of p53 was slightly diminished after Ti_0__.8_O_2_ nanosheet treatment when compared with control.

### 3.10. S-Nitrosylation in the Regulation of Stability of the Tetrameric p53 Protein–Protein Complex

**Figure 9:**
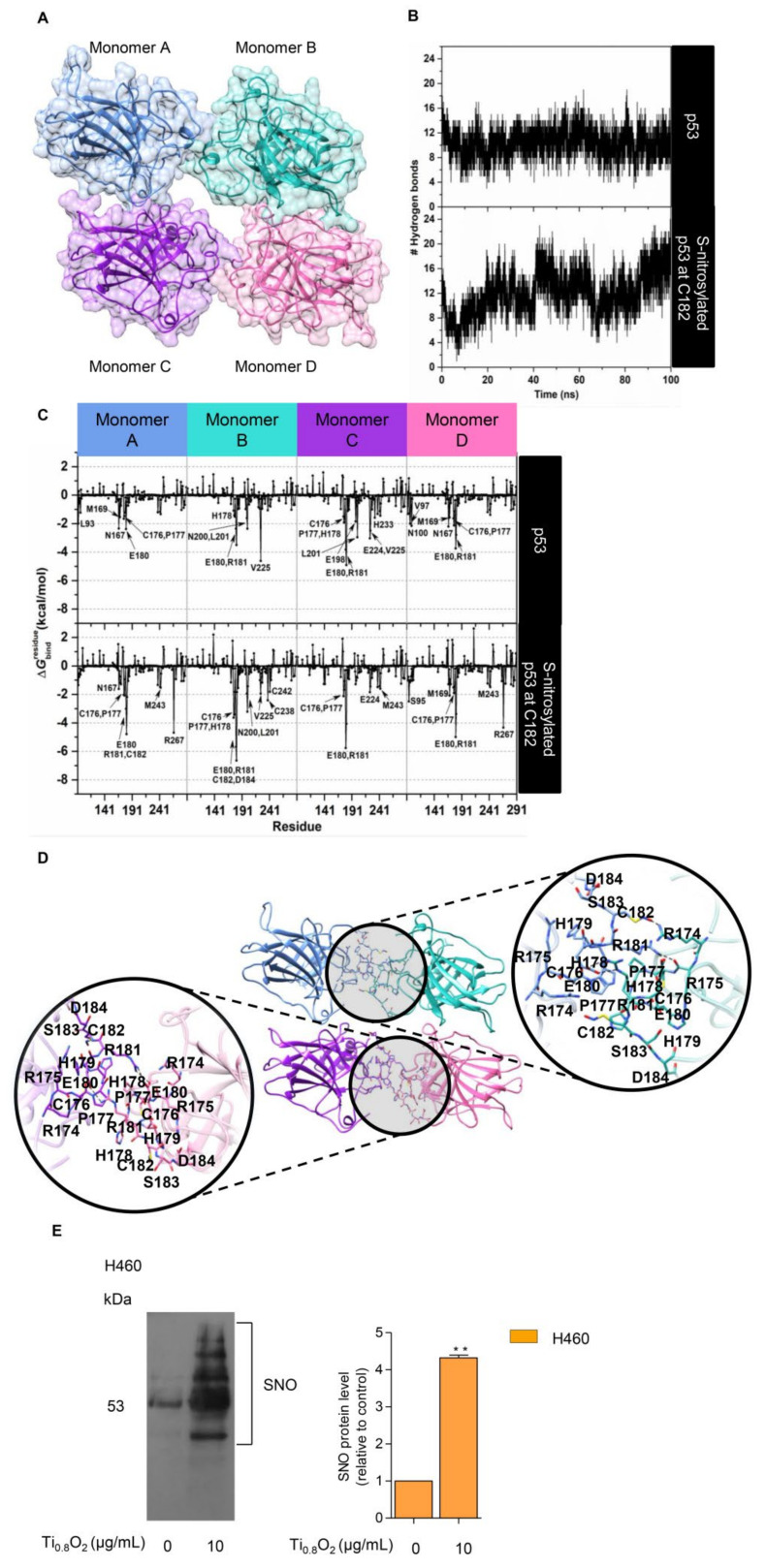
*S*-nitrosylation in the regulation of p53 stability. (**A**) Three-dimensional (3D) structure of the tetrameric p53 core domain without DNA bound (PDB ID: 3KMD); (**B**) time evolution of the total number of intermolecular hydrogen bonds formed between each monomer of the p53 core domain and its adjacent monomer; (**C**) the plot of (kcal/mol) of the p53 tetramer for the native form (top) and the C182 *S*-nitrosylation (bottom) system; (**D**) the representative 3D structure taken from the last MD snapshot of the *S*-nitrosylation system, with hydrogen bonds and electrostatic interactions represented by black dashed lines; (**E**) *S*-nitrosylated proteins determined by Pierce *S*-nitrosylation Western blot assay. Relative-to-control protein levels are reported (*n* = 3). ** *p* < 0.01 compared with the untreated control at the same time.

NO exerts its effects via the formation of *S*-nitrosylation of cysteine (Cys) residues. *S*-nitrosylation is a significant post-translational modification that affects p53 functionality [14]. *S*-nitrosylation of a single Cys within HDM2 inhibits p53 binding and thereby stabilizes p53 and activates p53-dependent transcription [38]. We tested whether peroxynitrite may directly control p53 by *S*-nitrosylation and the activation of p53. To evaluate the structural stability of the p53 core domain tetramer (Figure 9A), the number of intermolecular hydrogen bonds formed between each monomer at the protein–protein interface was monitored along with the simulation time. Note that a hydrogen bond is defined by the following geometric criteria: (i) the distance between the hydrogen bond donor and acceptor atoms is less than 3.5 Å, and (ii) the angle between D-H•••A is larger than 120°. The obtained results showed that there was an average of ~10 ± 2 hydrogen bonds steadily formed over the course of the simulation time (Figure 9B, top). This observation suggested that our simulation model was highly stable. Therefore, 100 equilibrated MD snapshots, extracted from the last 20 ns, were used for further analysis in terms of the $\Delta G_{\mathrm{bind}}^{\mathrm{residue}}$ calculation.

The $\Delta G_{\mathrm{bind}}^{\mathrm{residue}}$ value was then calculated to verify the crucial amino acids involved in protein binding at the interface region of each monomer. The total contributing energy from each amino acid for the protein-protein complex is shown in Figure 9C (top), where the positive and negative $\Delta G_{\mathrm{bind}}^{\mathrm{residue}}$ values are associated with protein destabilization and stabilization, respectively. It is noteworthy that only amino acids exhibiting a $\Delta G_{\mathrm{bind}}^{\mathrm{residue}}$ of <−1.5 kcal/mol were marked as the key binding residues. The results showed that the crucial residues (L93, N167, M169, C176, P177, and E180 for monomer A; H178, E180, R181, N200, L201, and V225 for monomer B; C176, P177, H178, E180, R181, E198, L201, E224, V225, and H233 for monomer C; V97, N100, N167, M169, C176, P177, E180, and R181 for monomer D) played a pivotal role in the tetrameric protein-protein stabilization. Based on this calculation and upon visual inspection, it can be assumed that the cysteine residue within the protein–protein interface, particularly C182, is most likely to be a critical residue, which would be expected to be related to the *S*-nitrosylation site and, consequently, would lead to an increase in protein stability. To clarify such a hypothesis, the influence of *S*-nitrosylation on the C182 residue of p53 upon its binding interaction with each monomeric p53 was investigated by means of MD simulation, as per the native p53 system. The simulation indicated that the total number of intermolecular hydrogen bonds between four monomeric proteins was slightly increased over the whole simulation, with an average value of ~12 ± 2 hydrogen bonds, particularly in the last 10 ns (90–100 ns), in which the number of hydrogen bonds was found to be up to ~20 (Figure 9B, bottom). This reflected that the *S*-nitrosylated C182 resulted in higher stability of the tetrameric protein–protein complex compared to the native p53. In addition, the occurrence of C182 *S*-nitrosylation appeared to induce the surrounding residues, located in the interface region, to bind more tightly to each other, especially residues 176–186 (Figure 9C (bottom) and Figure 9D). Among these amino acids, the lowest $\Delta G_{\mathrm{bind}}^{\mathrm{residue}}$ value (~−5 to −7 kcal/mol) was observed for residue R181, most likely owing to the indirect stabilizing effect of the *S*-nitrosylation at C182. This was probably one of the reasons why the *S*-nitrosylation culminated in the higher stability of p53, as observed in the experimental data.

To confirm the effect of such a nanosheet in the induction of *S*-nitrosylation in the cells, we further evaluated the induction of *S*-nitrosylated proteins in response to Ti_0__.__8_O_2_ nanosheets. Specific *S*-nitrosylated protein detection was done using the Pierce *S*-nitrosylation Western blot kit assay, as described in Section 2. The results revealed that *S*-nitrosylated proteins were dramatically increased in the Ti_0__.8_O_2_-treated cells compared with the non-treated control group (Figure 9E). Taken together, the findings highlight the impact of Ti_0__.8_O_2_-induced peroxynitrite in promoting *S*-nitrosylation and increasing the stability of the tetrameric p53 protein-protein complex, which is responsible for apoptosis cell death in NSCLC.

## 4. Discussion

Lung cancer remains the major cause of cancer death worldwide. Nowadays, nanomaterials are showing remarkable potential to aid the diagnosis and treatment of cancer by enabling the more effective targeting of tumors [39]. Previous studies have revealed that nanomaterials can selectively sink into solid tumors, whereby they increase the bioavailability and decrease the toxicity of the encapsulated cytotoxic agents [39,40]. Ti_0__.8_O_2_ (an emerging 2D analog of TiO_2_) nanosheets can be derived from the potassium zinc titanate precursor K_0__.8_Zn_0__.4_Ti_1__.6_O_4_. The planar surface and functional motifs of such 2D inorganic nanosheets can be modified using a surface engineering process involving chemical bonding or physical adsorption [22], which facilitates applications in physiological environments, through, e.g., their biostability improvement, site-specific targeting capability, and multiple theranostic functions to facilitate oncological applications [18,40,41].

Although TiO_2_ nanoparticles and nanotubes have been extensively investigated for their possible applications [41,42], to the best of our knowledge, there have been limited studies on Ti_0__.8_O_2_ nanosheets. Consequently, here, we show for the first time that Ti_0__.8_O_2_ nanosheets can distinctively induce anti-cancer activity in human non-small cell lung cancer cells and advanced lung cancer cells from patients. This effect was demonstrated in several lung cancer lines in comparison to common chemotherapeutic drugs used in lung cancer patients. The Ti_0__.8_O_2_ nanosheets significantly increased cancer cell death in a concentration-dependent manner (Figure 2). Moreover, the Ti_0__.8_O_2_ nanosheets also mediated apoptosis in lung cancer cells in a concentration-dependent manner (Figure 2I–N). Moreover, SEM analysis revealed that 10 μg/mL Ti_0.8_O_2_ nanosheets altered cell morphology, while TEM analysis for the characterization of the Ti_0__.8_O_2_ nanosheets in cells showed that the Ti_0__.8_O_2_ nanosheets could appropriately disperse into H460 cells more easily than in DP cells (Figure 3B). A previous study showed that various types of nanoparticles (NPs), such as copper oxide nanoparticles, could be used to induce anti-cancer activity in cancer cells [43]. Next, we further examined whether the Ti_0__.8_O_2_ nanosheets could induce cell apoptosis in H460 and A549 cells. We found that the treatment of lung cancer cells with Ti_0__.8_O_2_ nanosheets resulted in a significant induction of p53, which may, at least in part, play a role in Ti_0__.8_O_2_ nanosheet-mediated apoptosis (Figure 3C,D). Consistent with our findings, a previous study showed that FePt/GO nanosheets suppressed proliferation and induced apoptosis in H1975 cells, and silver nanoparticles induced apoptosis in human colon cancer cells mediated by p53 [44,45].

Most cancers possess aberrant or disrupted p53 pathways. Intervention to restore or enhance p53 activity is a promising cancer treatment strategy [46]. Interestingly, in this study, we further confirmed that Ti_0__.__8_O_2_ nanosheets also had cytotoxicity in patient-derived primary lung cancer cells, with a lower IC_50_ compared to some other first-line chemotherapeutic drugs tested (Figure 4). In addition, we have provided supportive information explaining that the cancer cell selectivity of Ti_0__.__8_O_2_ nanosheets may be caused by the generation of superoxide anions, similar to findings in a previous study [20]. Another previous study suggested that the effect of nanosilver on apoptosis was via ROS generation and the JNK-dependent pathway [47]. In addition, aminoflavone (AF) has been demonstrated to cause selective cell death induction in breast cancer cells, with minimal toxicity to normal breast cells. Aminoflavone caused an ROS increase, which is linked with the activation of caspase 3 and apoptosis, which can be prevented by the pretreatment of the cells with N-acetyl-L-cysteine (NAC) [48,49]. However, the generation of ROS was not associated with lung cancer cell death when the cells were pretreated with NAC or GSH, and the cell viability of the cancer cells could not be reversed (Figure 5C,D). This result suggests that the Ti_0__.__8_O_2_ nanosheets may generate other ROS for inducing cell death. Therefore, we investigated the NO level in lung cancer cells because its activity could be associated with cell death, as previously described [16]. The results showed that the NO levels were increased in a concentration-dependent manner (Figure 6A,C). Additionally, co-treatment of Ti_0.8_O_2_ nanosheets with PTIO (NO scavenger) or MnTBAP (superoxide anion inhibitor) inhibited Ti_0__.__8_O_2_ nanosheet-induced cell death by increasing cell viability. Much evidence has demonstrated that the direct toxicity of NO is modest but can be greatly enhanced by reacting with the superoxide anion to form peroxynitrite (ONOO^−^), which can directly damage DNA and attenuate DNA repair [50,51].

p53 is a tumor suppressor gene, regulating apoptosis and cell cycle arrest in cells that have damaged DNA. We found that the generation of peroxynitrite after treatment with Ti_0__.8_O_2_ nanosheets upregulated the expression of p53-mediated apoptosis (Figure 7). The data support the hypothesis that peroxynitrite contributes to the tumorigenic properties of p53 mutations. Peroxynitrite was found to induce mitochondrial permeability transition changes and promote apoptosis in cell-free systems containing mitochondria [52]. The degradation of p53 in normal cells is regulated through ubiquitination by the E3 ubiquitin ligase Mdm2 [53]. In this study, we found that under Ti_0__.8_O_2_ nanosheet treatment, the half-life of p53 was dramatically increased. The cycloheximide-based assay showed that the half-life of p53 in response to 10 µg/mL Ti_0__.8_O_2_ nanosheets was about 60 min in comparison to 30–40 min in non-treated control cells (Figure 8A–C). After applying the selective proteasome inhibitor (MG132), we monitored the levels of the p53-ubiquitin complex and found that the formation of the complex was dramatically decreased in the Ti_0__.8_O_2_ nanosheet-treated cancer cells (Figure 8D,E). Here, we have revealed novel information regarding the role of reactive nitrogen species, especially peroxynitrite, in the regulation of p53 tetramerization. Our results showed that when the cells were exposed to the nanosheets, the intracellular level of peroxynitrite was highly upregulated (Figure 6). Concomitantly, increased p53 was detected (Figure 7A–C) with the decrease in the p53-ubiquitin complex (Figure 8D,E), implying that the upregulation of p53 occurs as a result of preventing its degradation process.

Peroxynitrite is considered an important biological inducer via its direct interaction with protein in *S*-nitrosylation. *S*-nitrosylation is a rapid interaction wherein NO is attached to a thiol moiety of the target protein, forming *S*-NO at the cysteine amino acid [54]. More than 1000 proteins have been found as the targets of *S*-nitrosylation [55], and it was noted that such protein modification resulted in a profound alteration of protein–protein interaction, protein function, protein localization, and protein stability [56]. Cellular stress, such as through cisplatin (CDDP) treatment, activates and stabilizes p53 via phosphorylation at the sites of Ser 15 and/or Ser 20, subsequently blocking p53–Mdm2 interaction and suppressing p53 degradation [57].

**Figure 10:**
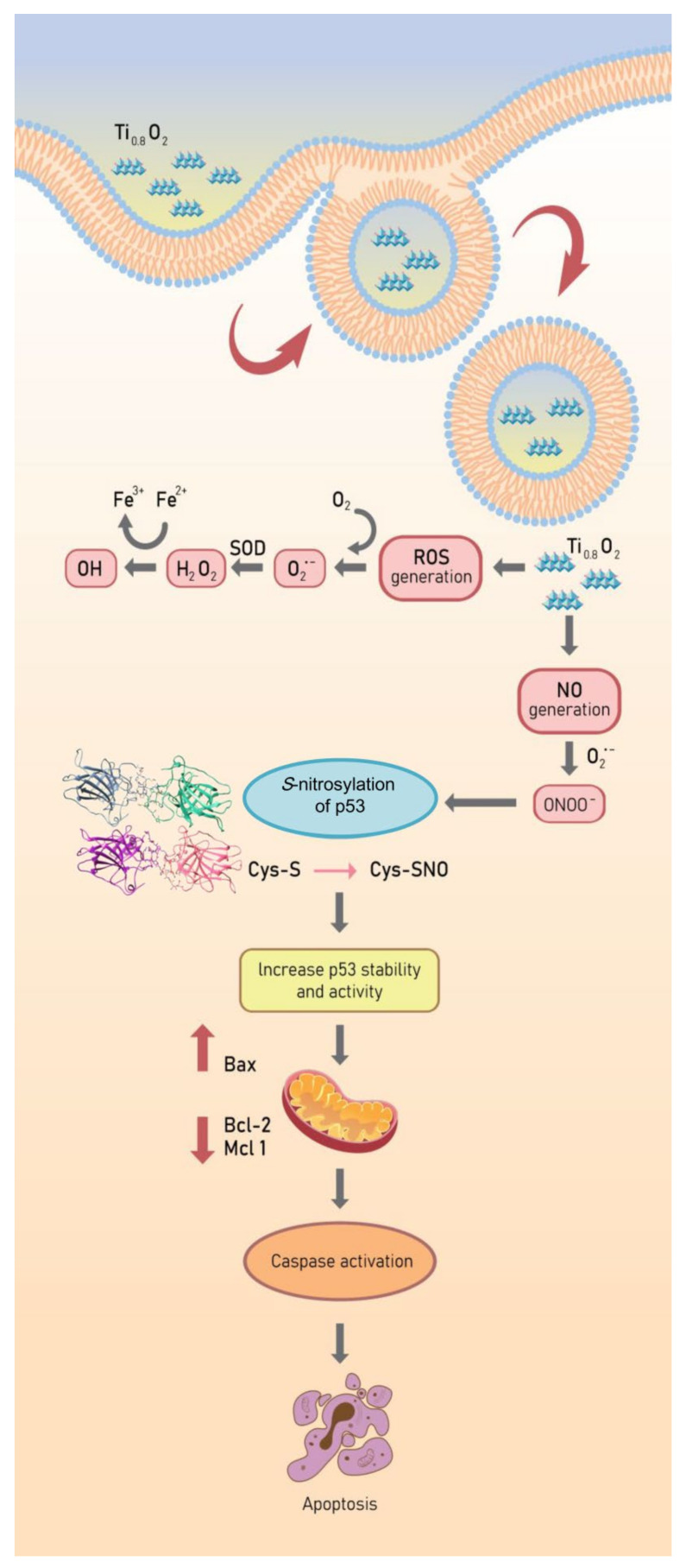
Schematic diagram of Ti_0__.8_O_2_ nanosheet-mediated peroxynitrite generation that was associated with apoptosis via p53 upregulation in non-small cell lung cancer.

The p53 protein plays a crucial role as a transcription factor regulating the expression of many proteins controlling cell arrest and apoptosis [58]. The p53 protein contains a tetramerization domain (p53TD) and a DNA-binding domain (p53DBD) which are important for protein functions [59]. Interestingly, evidence indicates that the activity of the p53 protein is highly dependent on tetrameric complex formation and complex stability [60]. Therefore, molecules capable of stabilizing the tetrameric form of the proteins could be promising therapeutical tools. Furthermore, we report additional studies on the role of hydrogen bond interactions in protein stability and the key binding residues of p53 to direct the effect of *S*-nitrosylation (Figure 9). In globular proteins, there are intermolecular hydrogen bonds between the protein and water molecules and between water molecules that are bound with the proteins [61]. Here, we used computational tools to predict the point of *S*-nitrosylation on the p53 protein and found that peroxynitrite may directly control p53 by *S*-nitrosylation to stabilize the tetrameric structure of this protein. To estimate the contribution of these hydrogen bonds to the conformational stability of a protein compared with that of the native p53 and *S*-nitrosylation of p53 [62], we investigated the relationship between *S*-nitrosylation and the increase in p53 stability. We identified the H-bond intermolecular interactions between a monomer of native p53 compared to its *S*-nitrosylation form and found higher stability of the tetrameric protein–protein complex in comparison to the native p53, especially regarding the reactivity of the cysteine at residue 182 in p53. The high reactivity of specific cysteine thiol groups in p53 is likely important for the regulation of p53 and its degradation pathways [38]. Moreover, peroxynitrite has been shown to activate the opening of mitochondrial pores that release cytochrome c into the cytoplasm [63]. According to our results, we found that peroxynitrite induces p53 stability and increases the activation of Bax and, subsequently, caspase 3. These changes are all hallmarks of cell death (Figure 10). Furthermore, the nanosheets were shown to generate peroxynitrite in aggressively driven mechanisms, including the process for the *S*-nitrosylation of p53 for protein stabilization. This novel finding on the role of Ti_0__.__8_O_2_ nanosheets in p53-mediated apoptosis may have important implications in cancer treatment.

## 5. Conclusions

In conclusion, our present study, for the first time, provides information on the effect of Ti_0__.8_O_2_ nanosheet-induced apoptosis through a molecular mechanism involving peroxynitrite generation. After treatment with Ti_0__.8_O_2_ nanosheets, it may directly control p53 by *S*-nitrosylation to stabilize the tetrameric structure of this protein. This reflects that the *S*-nitrosylation at C182 of p53 results in higher stability of the tetrameric protein–protein complex compared to the native p53. Therefore, the results of this study ingeminate the novel mechanism of action of nanomaterials for cancer therapy.

## Data Availability

Data are contained within the article.
